# Biofortified *Pleurotus* Species as Sustainable Protein Sources: Mineral Bioaccumulation and Nutritional Enhancement

**DOI:** 10.3390/molecules31122102

**Published:** 2026-06-15

**Authors:** Roberto A. Costa, Maria G. Leichtweis, Bruno Melgar, Pablo A. García, José Pinela, Carla Pereira

**Affiliations:** 1 CIMO, LA SusTEC, Instituto Politécnico de Bragança, Campus de Santa Apolónia, 5300-253 Bragança, Portugal; roberto.costa@ipb.pt (R.A.C.); maria.gabriela@ipb.pt (M.G.L.); bruno.melgarc@ipb.pt (B.M.); jose.pinela@iniav.pt (J.P.); 2 Departamento de Ciencias Farmacéuticas, Facultad de Farmacia, CIETUS-IBSAL, Campus Miguel de Unamuno, Universidad de Salamanca, 37007 Salamanca, Spain; pabloagg@usal.es; 3 National Institute for Agricultural and Veterinary Research (INIAV), I.P., Rua dos Lágidos, Lugar da Madalena, Vairão, 4485-655 Vila do Conde, Portugal

**Keywords:** fungal biotechnology, protein quality, sustainable food production, iron accumulation, selenium enrichment, agro-industrial residues

## Abstract

Fungi of the genus *Pleurotus* are increasingly studied not only for their ecological versatility and saprotrophic capabilities but also for their potential in biotechnological applications such as nutrient bioaccumulation. As sustainable alternatives to animal protein sources, *Pleurotus* species combine high nutritional value with the ability to grow on agro-industrial residues. This review explores the bioaccumulation potential of *Pleurotus* species of essential compounds of biotechnological interest, particularly selenium and iron, focusing on applications in sustainable nutrition and functional ingredient development. Notably, the substrate composition can nearly double protein content, and selenium-enriched mushrooms can reach up to 858 µg/g without compromising biological efficiency, depending on the dose and chemical form. Similarly, iron biofortification achieved up to 4176 µg/g in *P. pulmonarius* with minimal productivity loss. Among the species analysed, *P. ostreatus* and *P. eryngii* stood out for their productivity and nutritional quality, while *P. citrinopileatus* recorded the highest protein content at 34.7% dry weight. Overall, mineral biofortification of *Pleurotus* spp. emerges as a promising strategy to support sustainable food systems, address global micronutrient deficiencies, and expand the biotechnological use of edible fungi.

## 1. Challenges of Animal-Based Protein Production

Among the most pressing topics, the global demand for animal protein has a significant environmental impact, contributing to deforestation, biodiversity loss, and other environmental issues. According to FAO [1], the livestock sector directly occupies around 30% of the global ice-free terrestrial surface and accounts for 70% of all agricultural land and also contributes to greenhouse gas (GHG) emissions, energy consumption, and water usage [1,2]. Röös et al. [2] conducted a comprehensive review assessing the environmental impacts of meat production, including contributions to global warming, acidification, eutrophication, land use, primary energy consumption, and pesticide-related toxicity. Based on these data, the authors concluded that the carbon footprint reflects only part of GHG emissions related to meat production, mainly indicating direct and indirect emissions of CO_2_ and other gases. However, they noted that other significant environmental impacts, such as intensive land use (including the conversion of forests to pasture or forage crops), water consumption and pollution, soil degradation, and biodiversity loss, require better assessment and correlation. In other words, meat production generates a series of relevant environmental effects that require a broad, integrated approach to accurately assess its true impacts and reinforce the need for sustainable strategies that holistically minimise them. On the other hand, although some findings indicate the potential for substantial reductions in net GHG emissions in pasture-based systems under favourable conditions for soil carbon sequestration, these systems still cannot be considered ecologically efficient when compared to most alternative food production strategies [3].

Despite advances in global food production, protein-energy malnutrition (PEM) and inadequate protein intake remain significant public health concerns, particularly in low-income regions and among vulnerable populations such as children and older adults. A Global Burden of Disease (GBD)-based analysis reported approximately 14.8 million prevalent PEM cases worldwide in 2019 under a strict clinical definition [4], while modelling studies estimate that approximately 12.2% of the global population is currently at risk of protein deficiency under present dietary patterns and inequality conditions [5]. These projections may rise to 15.1% (~1.4 billion people) by 2050 due to demographic changes alone, with climate change-associated increases in atmospheric CO_2_ potentially placing an additional 148 million people at risk of protein deficiency. At the same time, the global population is projected to reach approximately 9.7 billion by 2050 [6], substantially increasing food and protein demand and intensifying pressure on current food production systems.

In parallel, global meat production is expected to more than double, from 229 million tonnes in 1999–2001 to 465 million tonnes by 2050 [1], driven by population growth and increased per capita consumption. This projected growth is likely to generate proportional environmental impacts, further intensifying challenges related to sustainability and the use of natural resources. The European Union currently aims to reduce its net domestic GHG emissions by at least 55% compared to 1990 levels, with the goal of achieving climate neutrality (i.e., net-zero GHG emissions) by 2050 [7]. Therefore, given the high levels of resource consumption and GHG emissions associated with meat production, it is imperative to find alternative ways to complement or replace animal protein in order to feed the world’s population while mitigating environmental and economic impacts. In an effort to promote circular economy practices in livestock production, Khanal [8] explored alternative feed resources such as insects, marine macroalgae, and freshwater plants and algae as nutrient sources for livestock, reducing feed costs and enhancing the sustainability of the sector. While Tuomisto and de Mattos [9] found auspicious results when comparing the environmental impacts of conventionally produced European meat with those of cultured meat, an in vitro production technology based on tissue engineering techniques. Their study found that the environmental footprint of alternative proteins was found to be substantially lower than that of conventional protein, with 78–96% lower GHG emissions, up to 99% less land use, and 82–96% less water use, depending on the type of meat being compared (beef, sheep, pork, or poultry).

## 2. Non-Animal Proteins: Global Trends and Consumer Shifts

The search for meat alternatives is driven not only by legislative and regulatory pressure but also by shifting consumption habits and socioeconomic factors influencing food choice and accessibility. On one hand, some consumers are adopting healthier diets and becoming increasingly aware of the environmental impact of livestock farming and animal welfare concerns [10]. On the other hand, significant food security challenges remain, especially in regions where malnutrition remains a critical issue. For many populations, meat remains an essential source of protein and nutrients, yet economic barriers often limit access to high-quality products. The high cost of meat makes it difficult for lower-income populations to afford a balanced diet, worsening social disparities and nutritional gaps [11,12,13,14].

According to de Araújo et al. [10], consumer perceptions of meat and processed meat products are influenced by concerns about animal welfare and sustainability. However, van Bussel et al. [15] pointed out that many consumers lack sufficient knowledge about food system sustainability, while Hartmann et al. [16] identified limited awareness of the environmental impact of food choices as a major barrier to more sustainable food consumption behaviours. Thus, improving consumer education and access to information on dietary health and sustainability is critical [10,15,16].

In this context, vegetarianism and veganism have emerged as dietary movements that exclude meat consumption while promoting sustainability and fuelling economic growth through the rapidly expanding alternative protein market. Vegetarianism is broadly defined as both a social identity and a dietary choice that excludes meat products, with varying degrees of adherence, such as lacto-vegetarians, ovo-vegetarians, and pescatarians. Veganism, on the other hand, extends beyond diet and represents a lifestyle that excludes all animal-derived products for ethical, environmental, and health reasons [17,18].

According to a 2018 report by Just Eat, a global online food delivery service, the demand for meat-free meals grew by a remarkable 987% in 2017 [19]. In the United States, two-thirds of the surveyed population reported a reduction in meat consumption, particularly red and processed meat [20]. These trends have spurred a rise in products and services targeting this demographic, as evidenced by market analysis from firms such as Mintel [21] and Baum & Whiteman International Food Consultants [22]. As the market rapidly expands, demand for innovative products continues to grow. For instance, according to these reports, 14% of all new products launched in Europe in 2015 were vegetarian or vegan.

Reduced consumption of animal-derived foods may increase the risk of nutritional deficiencies associated with lower intake of bioavailable iron, vitamin B12, zinc, iodine, calcium, omega-3 fatty acids, and nutritionally relevant proteins if dietary planning is inadequate [23,24,25]. Although dietary supplements are widely available and can effectively help prevent many of these deficiencies, their efficacy depends on appropriate formulation, absorption efficiency, consumer adherence, affordability, and regular nutritional monitoring [26,27]. In addition, the unnecessary or uncontrolled use of certain mineral supplements, including iron (Fe) and selenium (Se), may also pose health risks [28,29]. These limitations highlight the importance of developing naturally nutrient-dense alternative protein sources capable of simultaneously providing proteins and essential micronutrients within a food matrix as part of more sustainable dietary strategies.

## 3. The Role of Mushrooms as Emerging Non-Animal Proteins

Edible mushrooms have emerged as sustainable and nutritious alternatives to conventional protein sources. They are increasingly incorporated into food and nutraceutical products due to their rich nutritional profile and versatility. As alternative protein solutions gain global relevance, plant- and fungal-based foods continue to evolve. However, in terms of protein content and amino acid profile, mushrooms hold a clear advantage over traditional plant-based sources [30,31,32]. Mushrooms are a highly nutritious and valuable food source, not only in the Mediterranean diet but also globally, revered for their texture, flavour, and nutritional profile. Low in calories, carbohydrates, and sodium, mushrooms contain several vitamins, particularly B-group vitamins, including thiamine, with reported ranges from 0.35 mg to 7.8 mg per 100 g of dry weight (DW), niacin between 46.0 mg and 108.7 mg per 100 g DW, riboflavin within the range of 1.63 mg to 5.0 mg per 100 g DW, and vitamin C levels varying from 1.4 mg to 9.4 mg per 100 g DW [33]. Additionally, they are an excellent protein source, containing approximately 19 to 35% on a dry weight basis, and generally provide all nine essential amino acids [34,35]. Although some plant-derived protein sources, such as soy, may present higher protein contents, edible fungi offer distinct functional and technological advantages. *Pleurotus* mushrooms possess a naturally fibrous texture and umami-rich flavour profile that favour their incorporation into meat alternative products with lower processing requirements [30,36]. Furthermore, they can be cultivated on low-cost lignocellulosic agro-industrial residues and subjected to controlled mineral biofortification, supporting both circular bioeconomy strategies and the development of nutrient-dense alternative foods.

Mushrooms are indeed a good source of protein and dietary fibre, contributing significantly to the intake of essential vitamins and minerals [37]. Their low energy density makes them particularly beneficial for weight management, while their relative low level of purine is useful for persons suffering from metabolic disorders such as gout. Additionally, their very low sodium content makes them suitable for individuals with high blood pressure. From an orthomolecular perspective, mushrooms contain several key vitamins, enabling a significant part of the daily requirement to be covered by consuming mushrooms. They are also rich in potassium and phosphorus, another important orthomolecular aspect, and contain a high level of Se, which is regarded as an excellent antioxidant [38,39,40].

In addition to their impressive nutritional profile, mushrooms have shown significant potential in replacing meat in various products. In studies on meat extenders, plant ingredients such as legumes, cereals, tubers, and fruits have been widely used to replace up to 50% of meat, increasing product yield with minimal sensory modifications. Mushrooms, however, enable higher proportions of meat replacement, while contributing to a higher fibre content and improving the perception of salt, allowing sodium reduction without significantly altering the physical–chemical properties of the final product [30].

## 4. Bringing to Light Pleurotus Mushrooms as a Sustainable Non-Animal Protein Source

### 4.1. Edible Mushrooms

According to the definition proposed by Chang and Miles [39], mushrooms are macrofungi characterised by a distinct fruiting body, growing either epigeous (above ground) or hypogeous (underground), and are large enough to be seen with the naked eye and picked by hand. The organic materials from which the fungi obtain their food are called substrates. They process their food by secreting degradative enzymes that break down complex polymers in the biomass where they grow (such as lignin, cellulose, and hemicellulose) into simpler molecules, which are then absorbed to support mycelial growth and fruiting body formation. Unlike vascular plants, fungi do not have true roots and instead anchor themselves through an extensive network of hyphae that collectively form the mycelium, which also facilitates substrate colonisation, enzymatic breakdown, and nutrient absorption.

Mushrooms and fungi in general are extremely diverse and widespread globally. Estimates reported by Hawksworth [41] suggest that there are up to 9.9 million species of fungi on Earth, of which approximately 74,000 to 120,000 have been formally described, with a widely accepted working figure of around 1.5 million species. This gap underscores fungi’s vast untapped potential, especially for food security. Edible mushrooms represent a sustainable solution to malnutrition because they can be produced in large quantities in a short period of time and offer a higher amount of protein per unit area compared to traditional crops [42]. However, the successful selection of mushroom species for cultivation must account for multiple variables, including favourable sensory attributes matching consumer preferences, the local availability of suitable lignocellulosic substrates, the species’ mode of nutrition (e.g., saprotrophic, mycorrhizal, or parasitic), and the feasibility of replicating species-specific environmental conditions without requiring intensive technological or energy inputs [43,44].

The majority of cultivated edible mushrooms are saprotrophic, deriving their nutrition from the decomposition of non-living organic matter. This ecological role positions them as critical agents in biogeochemical cycles, particularly in the recycling of carbon and nitrogen from plant biomass, including wood, leaf litter, and agricultural by-products. Their saprotrophic nature also underpins their adaptability to cultivation on diverse lignocellulosic wastes, such as straw, sawdust, and agro-industrial residues [45]. In contrast, some edible species form obligate mutualisms with living plants (e.g., ectomycorrhizal taxa like truffles and chanterelles), while others adopt parasitic strategies, exploiting living hosts for nutrients. Although mycorrhizal and parasitic mushrooms are ecologically significant, contributing to forest dynamics and biodiversity, their cultivation remains challenging due to their complex symbiotic requirements or host dependencies.

### 4.2. Pleurotus

*Pleurotus* spp., commonly known as oyster mushrooms, are valued as functional foods for their flavour, aroma, rich nutrition and medicinal benefits. They are among the most commercially important edible fungi, cultivated extensively worldwide. Alongside *Agaricus bisporus* and *Lentinula edodes*, *Pleurotus ostreatus* ranks among the most widely cultivated edible mushroom species globally [46]. Together with *Lentinula*, *Auricularia*, *Agaricus*, and *Flammulina*, *Pleurotus* makes up about 85% of the global cultivated edible mushrooms supply, and alone contributes around 25% of global cultivated edible mushroom supply [47,48]. In economic terms, estimates indicate that the global *P. ostreatus* market was valued at US$ 5.54 billion in 2023, with projections suggesting an increase to US$ 7.35 billion by 2032, representing a compound annual growth rate (CAGR) of 3.2% over the forecast period [49]. These trends underscore *Pleurotus* rising economic role in the mushroom sector.

In addition to their economic importance, *Pleurotus* species are versatile saprotrophic fungi that can grow on a wide range of substrates, including both wood and agricultural bioresidues, bringing notable sustainability advantages. This genus comprises approximately 40 species distributed across tropical and temperate regions, where they play a crucial role in lignocellulose degradation through extracellular enzyme secretion [45]. In fact, their ability to colonise and degrade lignocellulosic material makes them ideal for the bioconversion of organic waste into useful resources. When grown in the forest, *Pleurotus*, like other mushrooms, decompose organic matter, recycle nutrients and support ecological balance [50]. Likewise, by growing on agricultural waste such as straw and husks, they not only generate a sustainable source of protein but also contribute to the reduction and valorisation of low-value biomass. It is estimated that approximately 998 million tonnes of agricultural residues, including rice straw, wheat straw, and other cereal by-products, are generated annually [51]. By using these crop residues as substrates, *Pleurotus* cultivation supports both waste recycling and food security, especially in low-resource areas. In addition, the spent substrates are used as fertiliser, animal feed, and for biogas production [52]. Although they generate CO_2_ during decomposition, they play a significant role in the carbon cycle, with the mycelium contributing vitally to carbon sequestration in the soil [50]. This dual role—as natural decomposers and as food producers—positions *Pleurotus* as an innovative solution for both environmental recovery and the diversification of non-animal protein sources. Moreover, this adaptability not only makes *Pleurotus* a promising candidate for sustainable mushroom production but also contributes to waste management and the circular economy by converting low-value biomass into high-value products.

The nutritional profile of *Pleurotus* mushrooms underscores their increasing global demand. They are an excellent source of protein and dietary fibre, containing essential and non-essential amino acids, notably lysine and leucine, often lacking in cereal-based diets [48]. Beyond their nutritional contributions, *Pleurotus* species exhibit bioactive properties, including antioxidant, immunomodulatory, and cholesterol-lowering effects, which further enhance their significance as functional foods [53,54]. Given their economic viability, ecological benefits, and health-promoting attributes, the cultivation and consumption of *Pleurotus* species continue to gain momentum on a global scale.

### 4.3. Pleurotus Ostreatus

**Figure 1:**
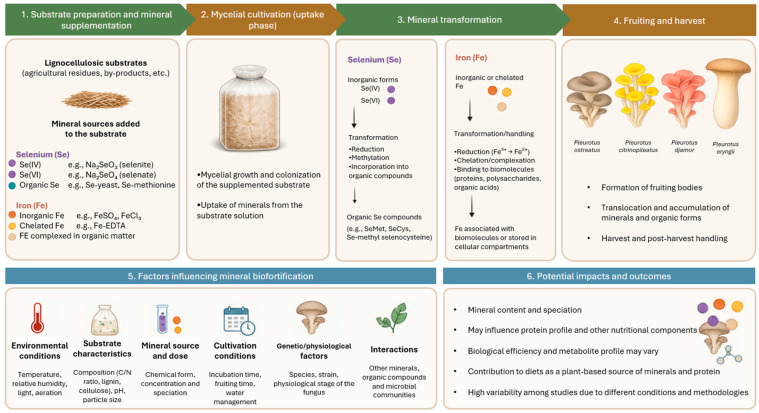
Schematic overview of Se and Fe biofortification in *Pleurotus* spp. from mineral-supplemented substrates to fruiting body accumulation and potential outcomes.

*Pleurotus ostreatus* is cultivated on a large scale, ranking third in the global market demand after button mushroom (*Agaricus bisporus*) and shiitake (*Lentinula edodes*) [48]. It has a distinctive morphology characterised by an irregular, asymmetrical cap that varies in shape from convex to flattened, often resembling an oyster shell (Figure 1). The surface of the cap is smooth, with colours ranging from white to grey or brown. The gills are widely spaced and change colour from white to creamy as they mature. The short, eccentric, or absent stipe contributes to the characteristic lateral attachment of the fruiting body. The flesh is firm but tender, with a mild, pleasant aroma, which contributes to its popularity as a cultivated edible mushroom [55].

Table 1 compiles data from multiple studies on *P. ostreatus* cultivation, highlighting significant variation in growth parameters, yield, and nutritional composition, primarily influenced by substrate type and the environmental conditions adopted. The substrates tested include agricultural, industrial, and vegetable waste, demonstrating species adaptability in breaking down various organic materials. Furthermore, factors such as temperature, relative humidity and lighting play a crucial role in the biological efficiency (BE) and protein content in the resulting mushrooms. It is possible to verify that nutrient-rich substrates, such as tea, coffee, and wheat straw residues, tend to generate higher BE values, often exceeding 100% [56,57,58], notably alfalfa pulp, achieved a BE of 166 ± 25% (Table 1) [58]. Similarly, the composition of the substrate directly influences the protein content, making it essential to choose substrates that guarantee high nutritional quality. For instance, Elkanah et al. [59] showed that strategic substrate selection can nearly double protein content, where supplementing palm oil waste and sawdust with wheat and rice bran resulted in protein levels ranging from 10.1 ± 0.1% to 19.1 ± 0.1% (Table 1).

Some studies implement mechanised control of growing conditions to standardise production [56,57,58,60,61], while others only monitor or estimate available environmental parameters [59,62,63,64,65,66]. This demonstrates that *P. ostreatus* can thrive in less controlled environments but also highlights a research gap in optimising these conditions to enhance yield and protein content.

**Table 1:** Cultivation parameters, biological efficiency, and protein content of *Pleurotus ostreatus* under different substrates.

Substrate	Incubation		Fruiting			Biological Efficiency (%)	Protein Content (% DW ^1^/FW ^2^)	Reference
	Temperature	Humidity	Temperature	Humidity	Light Intensity			
Waste tea leaves, sawdust, and rice straw	25 ± 2 °C	50–60%	25–30 °C	80–90%	150–200 lux	62–89	-	[61]
Corn cob, millet straw, and bamboo waste	25 °C	-	17–20 °C	Sprinkled with water twice a day	-	20.8 ± 0.4–50.2 ± 0.5	-	[63]
Acacia sawdust, corncob, and sugarcane bagasse	28 °C	60–70%	24 °C	≥90%	-	38.8–45.1	19.5–29.7/-	[67]
Coffee grounds, straw, and bottom ash	20 °C	50%	18–<28 °C	-	Daily natural light	-	-	[64]
Palm oil waste and sawdust supplemented with wheat and rice bran	25 ± 3 °C	65%	22–28 °C	Watered twice per day	Dark	-	10.1 ± 0.1–19.1 ± 0.1/-	[59,62]
Alfalfa pulp	24 °C	85%	18 °C	90%	324–444 lux	166 ± 25	20 ± 3/-	[58]
Coffee pulp or wheat straw	28 ± 1 °C	60 ± 5%	24 ± 5 °C	84 ± 6%	Daily natural light	50 ± 16–87 ± 41	-	[65]
Rice straw, wheat straw, corn cobs, and sugarcane bagasse	Room temperature	65–70%	Room temperature	Water sprayed three times a day	Dark	20.5–56.5	-/5.7–6.9	[66]
Olive pruning residues, spent coffee grounds, and fresh wheat straw	23–25 °C	80–90%	15 °C	88–90%	200 lux	95 ± 3–105 ± 27	-/2.160 ± 0.002–3.090 ± 0.002	[56]
Sunflower seed husk, soybean husk, and wheat straw	25 °C	85%	10–12 °C	85%	500 lux	16.7–37.2	-	[60]
Spent substrates and waste products of mushroom cultivation	26 ± 1 °C	80%	16.0 ± 1.0 °C	95%	700 lux	54 ± 8–133 ± 13	-	[57]

^1^ DW: dry weight; ^2^ FW: fresh weight.

As shown in Table 1, nutritional quality is a parameter that is infrequently considered by the authors, with fewer than half reporting protein content in oyster mushrooms. Nevertheless, Ahmed et al. [61] conducted a detailed analysis of amino acid composition, identifying eight essential and nine non-essential amino acids across all treatments. Notably, high concentrations were reported for histidine (ranging from 1.0 ± 0.3 to 2.4 ± 0.1 mg/100 g DW), glutamic acid (from 3.0 ± 0.2 to 4.5 ± 0.3 mg/100 g DW), and aspartic acid (from 1.7 ± 0.3 to 2.76 ± 0.09 mg/100 g DW). These results highlight both the nutritional value, since amino acid composition directly influences protein quality, and the variability in amino acid profiles under different cultivation treatments. In a context where mushrooms are being increasingly valued as a substitute for animal protein, exploring the influence of cultivation conditions on the protein content in *P. ostreatus* offers a potential pathway to improve the efficiency of its sustainable production and utilisation.

### 4.4. Other Pleurotus Species

Although *P. ostreatus* is the most widely cultivated and commercialised oyster mushroom, other species of the genus, such as *P. citrinopileatus*, *P. djamor*, and *P. eryngii* (Figure 1), are also gaining commercial importance.

*Pleurotus citrinopileatus*, commonly known as the yellow oyster mushroom, is valued for its vivid bright yellow cap and distinctive morphology. However, it is known for its initially bitter and pungent taste when lightly cooked, which may render it unpalatable to some consumers. Additionally, its delicate fruiting bodies are prone to damage during harvesting and packaging. Interestingly, when properly cooked until crisp, it develops a rich, nutty flavour reminiscent of cashews [68]. The global market for yellow oyster mushrooms was valued at approximately $ 1.4 billion in 2023, with projections indicating significant growth to $ 4.2 billion by 2033 [69]. This market still lags behind that of *P. eryngii*, valued at around $ 654.7 million and projected to grow at a CAGR of 6.5% over the forecast period of 2022–2027, according to the Industry ARC [70] report.

*Pleurotus eryngii*, known as the king oyster mushroom, is characterised by a convex to flat cap, beige to light brown in colour, and a robust, fleshy stipe significantly thicker than in other *Pleurotus* species. Unlike other oyster mushrooms, its gills are widely spaced and do not extend deeply into the stipe. This morphology enhances post-harvest durability, increasing its commercial appeal. It is also appreciated for its mild, umami flavour and firm, meaty texture, which resembles seafood or even white meat when prepared properly [71,72]. Meanwhile, *P. djamor*, also known as the pink oyster mushroom, exhibits vibrant colouration from light pink to red and is known for a milder, sweeter flavour. It is often used in dishes that require a more delicate taste. Its stripe is thinner, and its texture is softer compared to *P. eryngii* [73]. 

Under the cultivation conditions summarised in Table 2, the studies utilised more complex or enriched substrates, including *Ginkgo biloba* L. leaves, sugarcane and agave bagasse, and other agro-industrial residues. While the different *Pleurotus* species were incubated under relatively similar temperature conditions, more pronounced differences emerged during the fruiting phase. *P. eryngii* required lower fruiting temperatures [71,74,75,76] compared to *P. djamor*, indicating a preference for cooler fruiting conditions. Furthermore, the cultivation of *P. eryngii* often involves light regulation, including specific photoperiods and light intensities [71,74,75,76]. In contrast, light regulation has not been reported as essential for the successful cultivation of *P. djamor* and *P. citrinopileatus* [77,78,79,80,81].

Dharmasena et al. [77] conducted a detailed evaluation of culture conditions, spawn preparation, and cultivation of *P. djamor* strain PL01-White under several parameters. Optimal mycelial growth was observed at 25 °C (Table 2), with substantial inhibition at 35 °C. Fruiting occurred without requiring temperature shifts or substrate pre-treatment. Although yield was not significantly affected by substrate moisture content, higher moisture levels (65.5–67.7%) prolonged the harvesting period, with 65.5% identified as optimal. Likewise, no significant yield differences were observed under varying light intensities; however, illuminance levels of 500–5000 lux are recommended to ensure uniform mushroom colour and morphology [77].

As summarised in Table 2, Li et al. [74] implemented a two-day acclimatisation period in the cultivation of *P. eryngii* between the incubation and fruiting phases, emphasising the role of gradual environmental adjustments in promoting optimal development. During this intermediate stage, the fruiting chamber was maintained at 20 ± 2 °C, with 70–80% relative humidity and a CO_2_ concentration of 500–1000 ppm. In contrast, although less frequently studied, *P. citrinopileatus* demonstrated high BE (up to 74 ± 5%) and the highest protein content among the species evaluated, reaching up to 34.7 ± 0.8% DW when cultivated on a substrate composed of spent mushroom substrate, hydroponic vegetable roots, and wheat [81].

## 5. Biofortification of *Pleurotus* spp. With Essential Minerals: Focus on Selenium and Iron

Edible mushrooms, especially those of the genus *Pleurotus*, have attracted growing attention as functional foods due to their capacity to bioaccumulate and concentrate essential minerals [82]. In addition to Se and Fe, cultivated *Pleurotus* spp. naturally contain relevant concentrations of several macro- and microelements, although substantial variability exists among species, strains, substrates, and cultivation conditions. Furthermore, supplementation strategies have demonstrated that the concentrations of some of these elements can be significantly enhanced under controlled cultivation systems. Representative baseline ranges and examples of mineral-enriched cultivation approaches reported for *Pleurotus* spp. are summarised in Table 3.

This accumulation capacity is an intrinsic characteristic of these fungi, whose highly branched mycelial networks and efficient enzymatic system facilitate the uptake of nutrients from the substrate, including elements such as Fe [83,84,85], Se [86,87], zinc (Zn) [84,87], calcium (Ca) [88], and magnesium (Mg) [89]. Importantly, in the context of this review, such accumulation refers to controlled biofortification strategies using food-grade mineral supplementation and should not be confused with mycoremediation processes involving contaminated substrates or polluted environments. This phenomenon can be exploited in biofortification strategies aimed at increasing the micronutrient content in fruiting bodies, thereby helping to address mineral deficiencies in human populations [83]. The main stages involved in Se and Fe biofortification in *Pleurotus* spp., including mineral supplementation, uptake, transformation, and accumulation in fruiting bodies, are summarised in Figure 1.

Mushroom biofortification is an innovative, cost-effective, and scalable approach [82], especially in regions with a high prevalence of mineral deficiencies. In the case of Se, an essential micronutrient with antioxidant and immunomodulatory properties, several studies have shown that *P. ostreatus*, *P. eryngii*, and other species are capable of efficiently accumulating Se when supplemented in the substrate, either in organic forms (e.g., selenomethionine (SeMet) and selenocysteine (SeCys)) or inorganic forms (e.g., sodium selenate (Na_2_SeO_4_) and sodium selenite (Na_2_SeO_3_)) [90,91]. Regarding Fe, its bioavailability in mushrooms is a notable factor. *Pleurotus* spp. demonstrate a good capacity to accumulate Fe in their tissues and can be biofortified by supplementing the cultivation substrate with Fe based compounds, such as ferrous sulphate or Fe chelates [18,83].However, the comparison of mineral biofortification outcomes across studies is inherently constrained by substantial methodological heterogeneity, particularly regarding substrate composition and experimental design. Substrates differ not only in raw material but also in key physicochemical parameters such as carbon-to-nitrogen (C/N) ratio, lignocellulosic structure, and mineral-binding capacity, all of which directly influence fungal metabolism and nutrient uptake [58,59,61,76]. In general, lower C/N ratios promote nitrogen assimilation and metabolic activity, potentially enhancing the incorporation of minerals into fungal biomass, whereas higher C/N ratios, typical of lignocellulosic residues, may limit nutrient availability and reduce uptake efficiency [61,67]. Moreover, the relative proportions of lignin, cellulose, and hemicellulose affect substrate degradability and the release of low-molecular-weight compounds during fungal colonisation. The degradation of lignocellulose by *Pleurotus* spp. involves the production of extracellular enzymes and organic acids, which can modify substrate pH and promote metal solubilization through chelation and redox processes, thereby increasing the availability and mobility of elements such as Fe and Se [62]. However, these mechanisms are not selective for essential elements [92]. Depending on substrate composition, the same processes that enhance Fe and Se availability may also promote the co-accumulation of undesirable metals such as cadmium (Cd), lead (Pb), and mercury (Hg), with potential implications for food safety, as further discussed in Section 6. In addition, excessive concentrations of trace elements, including Copper (Cu) and Zn, may impair fungal metabolism, inhibit mycelial growth, and reduce biological efficiency, highlighting the importance of carefully controlling substrate composition and supplementation levels during biofortification [84,93].In addition to substrate-related factors, studies also differ in experimental design, including the chemical form of the mineral source, application method (solid vs. liquid supplementation), reporting units (e.g., mg/kg, mg/L, or mM), and the basis of expression (dry weight vs. fresh weight), while the absence of standardised reporting of biological efficiency (BE) further complicates interpretation. Therefore, the data presented in Table 4 and Table 5 should be interpreted as indicative of general trends rather than directly comparable values, and conclusions regarding optimal biofortification conditions must be considered within the context of each experimental system. These aspects are further discussed for Se and Fe in the following subsections.

**Table 3:** Reported baseline mineral composition and representative biofortification levels in cultivated *Pleurotus* spp.

Mineral	Reported Baseline Range in Cultivated *Pleurotus* spp. (mg/kg)	Representative Biofortified Concentration (mg/kg)	Representative Species/Examples	References
Calcium (Ca)	17.8–2240	5966	*P. ostreatus* supplemented with eggshell/snail shell	[94,95,96,97]
Copper (Cu)	0.9–54	981	*P. pulmonarius* cultivated on Cu-supplemented substrate	[94,95,96,98]
Iron (Fe)	0.3–775	See Table 4	Detailed Se biofortification discussed separately	[94,95,96]
Potassium (K)	10,970–56,710	Not specifically reported	-	[94,95]
Magnesium (Mg)	25.6–7830	14,550	*P. djamor* supplemented with Mg salts	[89,94,95,96]
Manganese (Mn)	0.1–47.5	Not specifically reported	-	[94,95,96]
Sodium (Na)	1–348	406	*P. djamor* cultivated on combined dairy manure-food waste digestate	[52,94,95]
Phosphorus (P)	4031–33,120	Not specifically reported	-	[94,95]
Zinc (Zn)	0.7–311	5230	*P. pulmonarius* treated with ZnO nanoparticles	[94,95,96,99]
Selenium (Se)	0.09–4.09	See Table 5	Detailed Se biofortification discussed separately	[95]

### 5.1. Selenium Biofortification

Se biofortification of *Pleurotus* spp. has demonstrated a wide range of effects on mushroom composition, biological efficiency, and nutritional potential. Several studies have reported enhanced Se accumulation in fruiting bodies, influenced by the chemical form of Se used, its concentration in the substrate, and the mushroom species cultivated, as shown in Table 4.

At the molecular level, Se biofortification in *Pleurotus* spp. is strongly dependent on its chemical form and subsequent metabolic transformation. Inorganic Se sources such as Se(IV) (as sodium selenite) and Se(VI) (as sodium selenate) are absorbed and incorporated into fungal metabolism through sulphur assimilation pathways, where they are enzymatically converted into organic Se species, including selenomethionine and selenocysteine [100,101]. These compounds can be non-specifically incorporated into proteins, replacing their sulphur analogues, which explains the association of Se with specific protein fractions observed in several studies [102]. Importantly, Se(IV) is generally more readily metabolised than Se(VI), which may explain its higher accumulation efficiency [86,91]. However, excessive Se concentrations can disrupt redox balance and induce metabolic stress, ultimately limiting uptake efficiency and affecting biological performance [100,101]. These mechanistic aspects help explain the variability observed across studies, particularly regarding differences in Se form, substrate concentration, and accumulation efficiency, as summarised in Table 3.

According to de Oliveira et al. [91], *P. ostreatus* showed a greater capacity to accumulate Se when grown on substrates supplemented with Se(IV) rather than Se(VI). In fact, Se concentrations in fruiting bodies were 3.8 and 2.6 times higher, respectively, at 12.8 and 51.2 mg/kg substrate loading under Se(IV) treatment. Additionally, Se mass accumulation per unit of fruiting body biomass confirmed this trend. Notably, *P. djamor* accumulated up to 1.7 times more Se than *P. ostreatus* under Se(IV) supplementation, with the optimal condition for both species identified as 51.2 mg/kg Se(IV) applied to an organic sugarcane bagasse-based substrate (Table 4). However, as highlighted by da Silva et al. [103], Se absorption by *P. ostreatus* is not strictly linear. Although the highest Se concentration in fruiting bodies (858 µg/g) was achieved with 102 mg/kg of Se-enriched substrates (Table 4), absorption efficiency declined at higher substrate concentrations. Substrates supplemented with 3.2 and 12.8 mg/kg Se enabled approximately 34% absorption of the added Se, whereas only 16% was absorbed at 51 mg/kg, likely due to toxicity effects or reduced bioavailability from excess sodium selenite. Despite this reduced efficiency at higher doses, the absolute Se accumulation in mushrooms increased with higher Se availability.

From a nutritional perspective, Se-enriched mushrooms may provide additional health benefits. Ji et al. [104] demonstrated that yeast-derived Se supplementation significantly increased the proportion of organic Se and its bioaccessibility in *P. eryngii* compared to mushrooms grown on Se(IV)-supplemented substrates. Furthermore, Se-enriched *P. eryngii* showed potential to promote gut health and mitigate lead toxicity, partly by increasing the abundance of lead-binding bacteria such as *Desulfovibrio*. These findings suggest that Se-yeast may be a viable alternative to inorganic forms for industrial Se enrichment. Among the four Se forms tested, (Se(IV), Se(VI), SeMet, and Se-yeast), Se-yeast resulted in the highest Se content in fruiting bodies, reaching 208 ± 5 µg/g at 60 mg/kg substrate loading (Table 4).

Protein composition is also affected by Se enrichment. Oliveira and Naozuka [105], evaluating Se(IV) supplementation at 6.4 to 25.6 mg/kg (Table 4), found that Se altered the solubility and distribution of proteins in both *P. ostreatus* and *P. djamor*, with Se mainly associated with albumins and glutelins. Additionally, Fasoranti et al. [96] reported an increase in total protein content in Se-fortified mushrooms, rising from approximately 10% in control groups to 18.2% in *P. ostreatus* and 16.4% in *P. pulmonarius*, a species from the same *Pleurotus* genus that shares similar morphological and nutritional characteristics.

Beyond protein enhancement, Se biofortification appears to stimulate the synthesis of bioactive compounds. Sravani et al. [106] reported significant increases in total soluble protein, phenolic compounds (including flavonoids), and antioxidant activity in Se-enriched *P. ostreatus* fruiting bodies. In this study, biofortification was achieved using substrates containing seleniferous soils, resulting in Se concentrations of 57 ± 7 µg/g in *P. eryngii* and 157 ± 8 µg/g in *P. djamor* (Table 4), without significantly affecting BE values. On the other hand, Fasoranti et al. [107] observed that non-fortified mushrooms exhibited higher BE than those fortified with 50 mg/kg Se(IV) (Table 4), possibly due to the metabolic stress induced by excessive Se levels. Similarly, Madaan et al. [93] noted that supplementation with inorganic Se and Zn salts enhanced the biochemical potential of *Pleurotus* spp., supporting their application in the development of functional foods and dietary supplements. Moreover, phenolic compounds and other naturally occurring metabolites in mushrooms may act synergistically to enhance the intestinal absorption of minerals such as Fe, thereby increasing the overall nutritional value of Se-enriched mushrooms [103].

Overall, available studies suggest that inorganic Se, particularly Se(IV), is more readily accumulated than Se(VI), likely due to its more direct incorporation into fungal metabolic pathways. Inorganic Se supplementation may result in the formation of organic Se species within fungal tissues, particularly selenomethionine (Se-Met), which has been identified as one of the major Se forms in enriched *P. ostreatus* [86]. The nutritional relevance of Se enrichment is therefore strongly dependent on Se speciation, as organic Se species are generally considered more nutritionally relevant than inorganic forms. In addition, Se(IV)-supplemented mushrooms have shown higher proportions of bioaccessible Se associated with low-molecular-weight fractions compared to Se(VI)-supplemented mushrooms [91]. However, most available studies report only total Se accumulation, without detailed characterisation of Se speciation, limiting direct conclusions regarding nutritional quality and bioavailability. While in vitro digestion studies suggest that Se from enriched mushrooms can be released in potentially bioaccessible forms, gastrointestinal availability may still be influenced by Se speciation and matrix interactions. However, in vivo and clinical evidence remains limited, preventing definitive conclusions regarding its nutritional efficacy [108].

Regarding dosage, most studies indicate a non-linear relationship between substrate Se concentration and accumulation, with moderate supplementation levels generally promoting higher uptake efficiency, while excessive concentrations may reduce biological efficiency (BE) due to metabolic stress. This suggests a trade-off between maximising Se accumulation and maintaining fungal productivity. In addition, the risks of Se overaccumulation must be carefully considered. Although a daily intake of 55 µg meets the recommended dietary allowances for adults [109], prolonged exposure to concentrations above the tolerable upper intake level (UL) of 400 µg/day [86] may pose health risks.

Therefore, high Se accumulation does not necessarily translate into improved nutritional value, and the reported results should be interpreted as indicative trends rather than definitive conclusions, particularly given the methodological heterogeneity across studies.

**Table 4:** Summary of Se fortification experiments conducted with *Pleurotus* spp.

*Pleurotus* Species	Se Form Applied ^1^	Se Load per (Dry Substrate) ^2^	Maximum Biological Efficiency (BE)	Maximum Se Concentration in Fruiting Bodies (Dry Mass) ^2^	Reference
*P. ostreatus*	Se(IV)	3.2–102 mg/kg	66% (at 12.7 mg/kg)	858 µg/g (at 102 mg/kg)	[103]
*P. ostreatus*	Se(IV)	6.4–25.6 mg/kg	24% (at 12.8 mg/kg)	48 ± 2 µg/g (at 25.6 mg/kg)	[105]
*P. ostreatus*	Se(IV) and Se(VI)	12.8 and 51.2 mg/kg	-	156 ± 4 µg/g (at 51.2 mg/kg of Se(IV))	[91]
*P. ostreatus*	Se(IV)	50 mg/kg	113.7%	52 ± 3 µg/g	[107]
*P. ostreatus*	Se(IV) and Se(VI)	5 mL of 1 g/L solution per kg of substrate (wet basis; dry matter not reported)	-	94 ± 1 µg/g (of Se(VI))	[86]
*P. ostreatus*	Seleniferous soils	22.34 mg/kg	67 ± 1%	124 ± 8 µg/g	[106]
*P. eryngii*	Se(IV), Se(VI), SeMet and Se-yeast	1–80 mg/kg	-	208 ± µg/g (at 60 mg/kg of Se-yeast form)	[104]
*P. eryngii*	Seleniferous soils	22.34 mg/kg	30 ± 2%	57 ± 7 µg/g	[106]
*P. djamor*	Seleniferous soils	22.34 mg/kg	30 ± 2%	157 ± 8 µg/g	[106]
*P. djamor*	Se(IV) and Se(VI)	12.8 and 51.2 mg/kg	-	196 ± 5 and 205 ± 4 µg/g (at 51.2 mg/kg, respectively)	[91]
*P. djamor*	Se(IV)	6.4–25.6 mg/kg	27% (at 25.6 mg/kg)	76 ± 2 µg/g (at 25.6 mg/kg)	[105]
*P. pulmonarius*	Se(IV)	50 mg/kg	133.1%	59 ± 3 µg/g	[107]

^1^ Se(IV): sodium selenite (Na_2_SeO_3_), Se(VI): sodium selenate (Na_2_SeO_4_), SeMet: selenomethionine, and Se-yeast: selenium-enriched yeast. ^2^ the equivalence of 1 μg/g = 1 mg/kg was applied throughout the table.

### 5.2. Iron Biofortification

Fe biofortification is most commonly achieved by adding Fe salts to the cultivation substrate. At the molecular level, Fe uptake involves both passive absorption and biologically mediated processes. Fe is typically absorbed in its soluble forms (Fe^2+^ and Fe^3+^), with its bioavailability strongly influenced by substrate conditions such as pH, redox potential, and ligand availability. Fungi may facilitate Fe acquisition through reductive mechanisms and the secretion of siderophore-like compounds that enhance Fe solubility [110,111,112]. Once internalised, Fe can bind to fungal cell wall components such as chitin and glucans, or associate with intracellular proteins and enzymes, contributing to its accumulation in fruiting bodies [110,113]. However, excessive Fe concentrations may induce oxidative stress via Fenton-type reactions, negatively affecting fungal metabolism and growth performance [114,115].

The most commonly used compounds include ferric chloride hexahydrate (FeCl_3_·6H_2_O), ferrous sulphate heptahydrate (FeSO_4_·7H_2_O), and the ferric complex FeHBED (ferric potassium complex of N,N′-bis(2-hydroxybenzyl)ethylenediamine-N,N′-diacetic acid), a stable, water-soluble Fe chelate [83]. These forms differ significantly in their chemical behaviour. FeSO_4_ provides readily available Fe^2+^ but is prone to oxidation; FeCl_3_ supplies Fe^3+^, though it may have limited solubility depending on substrate conditions; and FeHBED maintains Fe in a soluble, chelated form, enhancing its stability and availability for uptake.

In general, increasing Fe concentrations in the substrate results in greater Fe accumulation in the fruiting bodies, although this relationship is not always linear, likely due to variations in Fe bioavailability and species-specific physiological tolerance mechanisms. In the study by Budzyńska et al. [83], *P. eryngii* and *P. ostreatus* showed significant increases in Fe content in the fruiting bodies when grown with 50 mM of each Fe salt. The highest level was observed in *P. ostreatus* treated with FeHBED (up to 18 ± 1 µg/g), while *P. eryngii* showed statistically similar values across the three salts (16.3–16.8 µg/g). These results are presented in Table 5; however, the lack of BE data limits interpretation regarding productivity impact. For *Pholiota nameko*, which was also evaluated in the same study and cultivated under the same conditions as *P. eryngii*, the most effective Fe accumulation (up to 285% above the control) was achieved with FeCl_3_·6H_2_O at 1680 mg/kg. On the other hand, *P. pulmonarius* presented the highest Fe content among all fruiting bodies (Table 5): 4176 ± 80 µg/g after substrate supplementation with 500 mg/kg of FeSO_4_ [84]. Notably, this enrichment did not compromise BE, which remained at 47.7%, similar to the control (48.8%). This data suggests that, depending on the species, high Fe supplementation levels may be well tolerated. In contrast, some studies found that low concentrations of FeSO_4_, such as 0.8 mg/kg [116], failed to enhance Fe accumulation in mushrooms, possibly due to poor bioavailability of the mineral, influenced by substrate pH, aeration, and compound solubility.

Yokota et al. [117] reported that *P. ostreatus* supplemented with 100 and 500 mg/kg FeSO_4_ resulted in a 2.4 to 4.4 times increase in Fe concentration in mushrooms (up to 478.7 µg/g, Table 5), but BE values decreased markedly (by 18.8% and 94.3%, respectively) compared to the control. Similarly, Ogidi et al. [118] demonstrated the inhibitory effect of Fe at high concentrations (0–100 mg/L of Fe as FeSO_4_) on mycelial growth and biomass production in several *Pleurotus* species. Under these conditions, the Fe content in the fruiting bodies ranged from 37.8 to 96.6 µg/g, reinforcing that accumulation is influenced not only substrate Fe concentration but also on its bioavailability and species-specific physiological tolerance.

At lower concentrations, Fe biofortification may be limited by poor solubility or complexation under specific substrate conditions. Studies using very low Fe concentrations, such as that of Vieira et al. [116], who applied 0.8 mg/kg of FeSO_4_ (Table 5) in coffee husk substrate for *P. ostreatus* cultivation, led to Fe contents lower than those in the control (111.5 vs. 149.1 µg/g, respectively), indicating the existence of a minimum threshold for effective biofortification.

Fe biofortification also affects the profile of bioactive compounds in mushrooms. Budzyńska et al. [83] reported significant changes in phenolic and organic acid contents in *P. ostreatus* and *P. eryngii* under different Fe salts and concentrations. In *P. ostreatus*, the phenolic acid content increased with 168 mg/kg FeCl_3_ but decreased at higher concentrations or with other salts. Regarding organic acids, *P. eryngii* showed lower overall production, while *P. ostreatus* had greater variability, including an increase in malic acid with FeHBED. Additionally, the presence of Fe in the substrate has been shown to promote the accumulation of other minerals, such as Ca, K, Mg, Mn, Na, P and S, and the addition of Ca also influenced Fe absorption, indicating potential synergistic or antagonistic interactions between the elements [92].

In general, Fe accumulation in *Pleurotus* spp. appears to be influenced by both the chemical form and solubility of the Fe source. However, most available studies have evaluated FeSO_4_ supplementation, while only a limited number of studies directly compared different Fe sources, making it difficult to establish consistent conclusions regarding the most effective form for Fe accumulation. Similarly, increasing Fe concentration in the substrate does not necessarily result in a proportional increase in accumulation, as uptake efficiency may be influenced by substrate characteristics and fungal physiological responses to supplementation. Some studies suggest that elevated Fe supplementation levels may negatively affect biological efficiency (BE), although available data remain limited.

The nutritional relevance of Fe enrichment depends not only on total accumulation but also on its chemical speciation and interactions with fungal biomolecules [112]. Fe may associate with proteins, polysaccharides (e.g., chitin), and phenolic compounds, which can influence its solubility and gastrointestinal accessibility [111,113]. From a nutritional perspective, Fe^2+^ is generally considered more readily absorbed than Fe^3+^, although strong complexation with fungal matrix components may either enhance stability or limit bioaccessibility depending on the system [112]. However, most available studies report only total Fe accumulation, without detailed characterisation of Fe speciation or bioaccessible fractions, limiting direct conclusions regarding its nutritional quality and effective absorption. Although in vitro studies suggest that mushroom matrices may support Fe bioaccessibility [119], clinical evidence remains scarce, and the relationship between total accumulation and effective absorption is not yet fully established [120]. Given the variability in experimental conditions and reporting methods, these findings should be interpreted as indicative trends rather than universally applicable outcomes.

**Table 5:** Summary of Fe fortification experiments in *Pleurotus* spp.

*Pleurotus* Species	Fe Form Applied ^1^	Fe Load per Dry Substrate	Maximum Biological Efficiency (BE)	Maximum Fe Concentration in Fruiting Bodies (Dry Mass)	Reference
*P. eryngii*	FeCl_3_, FeSO_4_, or FeHBED	168, 336, 1680 mg/kg	-	17 ± 2, 17 ± 1, and 16 ± 1 µg/g (at 1680 mg/kg, respectively)	[83]
*P. ostreatus*	FeCl_3_, FeSO_4_, or FeHBED	168, 336, 1680 mg/kg	-	18 ± 1 µg/g (at 1680 mg/kg of FeHBED)	[83]
*P. pulmonarius*	FeSO_4_	500 mg/kg	47.8%	4176 ± 80 µg/g	[84]
*P. ostreatus*	FeSO_4_	0.8 mg/kg	-	111.5 µg/g	[116]
*P. ostreatus*	FeSO_4_	100–500 mg/kg	29.7% (at 100 mg/kg)	478.7 µg/g (at 500 mg/kg)	[117]
*P. ostreatus*	FeSO_4_	500 and 1000 mg/kg	-	88.5 µg/g (at 1000 mg/kg)	[118]
*P. djamor*	FeSO_4_	500 and 1000 mg/kg	-	74.2 µg/g (at 1000 mg/kg)	[118]
*P. pulmonarius*	FeSO_4_	500 and 1000 mg/kg	-	96.6 µg/g (at 500 mg/kg)	[118]

^1^ FeCl_3_: ferric chloride; FeSO_4_: ferrous sulphate; FeHBED: ferric potassium complex of N,N′-bis(2-hydroxybenzyl)ethylenediamine-N,N′-diacetic acid.

## 6. Contaminant Bioaccumulation and Food Safety Risks

Mushrooms possess a remarkable ability to absorb essential micronutrients like Se and Fe from their growth substrates. However, this same trait makes them highly prone to accumulating environmental contaminants, including heavy metals and pesticide residues. The extent of bioaccumulation varies among species and is influenced by substrate composition and local environmental conditions. For instance, *P. ostreatus* and *A. bisporus* demonstrate high uptake efficiency for heavy metals such as Cd, Pb, and Hg [121,122,123]. In some wild mushrooms collected from contaminated areas, Pb and Cd levels have been reported to pose a significant health risk to humans, with Pb levels found to be higher in saprophytic fungi compared to mycorrhizal species [124]. Furthermore, among a total of 354 edible fungi investigated by Li et al. [125], the fungicide carbendazim had the highest detection rate (70.9%), followed by the insecticide acephate (13.0%) and the fungicide procymidone (7.3%), which should be monitored during edible fungi production. These findings underscore the need for preventive strategies to minimise contamination risks during both wild harvesting and commercial cultivation of mushrooms. Particular attention should be given to the use of agro-industrial bioresidues, as they may introduce hazardous substances into the fungal growth environment. To limit contamination transfer into edible fruiting bodies, strict regulation of substrate selection and formulation is essential. Such measures are critical to ensuring food safety and protecting consumer health. In response to these concerns, the European Commission has established maximum levels of Cd and Pb in certain cultivated mushrooms, including *P. ostreatus* and *A. bisporus*, with limits of 0.30 mg/kg fresh weight for Pb and 0.20 mg/kg fresh weight for Cd under Regulation (EU) 2023/915 [126].

From a nutritional and toxicological perspective, the balance between beneficial and excessive intake must also be considered. Se, for example, is an essential micronutrient but presents a narrow safety margin, with a recommended dietary allowance (RDA) of approximately 55 µg/day and a tolerable upper intake level (UL) of 400 µg/day for adults [86,109]. Similarly, excessive Fe intake may result in adverse effects, with upper intake levels in the range of 40–45 mg/day for adults proposed by international nutritional authorities (e.g., EFSA and the U.S. Institute of Medicine) [127].These thresholds highlight the importance of controlling mineral concentrations in biofortified mushrooms to ensure safe consumption. Overall, the successful application of biofortification in *Pleurotus* spp. requires a balanced approach that integrates nutritional enhancement with rigorous safety assessment. This includes careful substrate selection, controlled mineral supplementation, and systematic monitoring of both target and non-target elements in the final product. From a practical standpoint, the feasibility of biofortified mushrooms as functional foods depends not only on their nutritional benefits but also on their compliance with food safety regulations and their capacity to maintain contaminant levels within safe limits.

## 7. Contribution of Biofortified Mushroom Production to the UN Sustainable Development Goals

The challenge of feeding the world’s growing population while respecting planetary boundaries lies at the heart of the 2030 Agenda for Sustainable Development. This ambitious framework, articulated through 17 Sustainable Development Goals (SDGs), calls for innovative approaches to food production that simultaneously address nutrition, equity, and environmental sustainability. In this context, the biofortification of *Pleurotus* species to develop protein-rich food alternatives offers a promising solution, especially when integrated with controlled environment agriculture (CEA) and vertical farming systems. These systems allow year-round production, efficient space use, optimal resource utilisation, low overall carbon footprint, automation and traceability, and the use of low-cost, renewable materials, thus creating synergies across multiple SDGs.

The development of biofortified mushrooms enriched in essential minerals such as Fe and Se aligns most directly with SDGs 2 (Zero Hunger) and 3 (Good Health and Well-being). By increasing concentrations of these essential minerals in mushroom fruiting bodies, this approach may contribute to widespread micronutrient deficiencies associated with anaemia, immune dysfunction, and cognitive impairments. These benefits are further amplified by indoor cultivation in vertical modules or racks, which ensure consistent yields regardless of external climate conditions, thereby enhancing food security, particularly in regions vulnerable to climate change and nutritional shortages. Moreover, controlled environment production systems contribute to SDG 9 (Industry, Innovation, and Infrastructure) through the adoption of automation and precision agriculture technologies that optimise substrate and resource use and minimise waste.

From an environmental perspective, edible mushroom cultivation in controlled environment rooms and environmental chambers supports SDG 12 (Responsible Consumption and Production) and SDG 13 (Climate Action), particularly through the use of agro-industrial bioresidues, contributing to waste valorisation and circular bioeconomy strategies. Additionally, vertical farming may reduce food miles by enabling production close to urban centres. Within the broader context of alternative protein systems, fungal-derived products such as mycoprotein have been associated with substantially lower greenhouse gas emissions compared to conventional beef production. Life cycle assessment studies report emissions of approximately 0.73 kg CO_2_ eq/kg for mycoprotein-based products, whereas beef production systems may range from 10.2 to 37.6 kg CO_2_ eq/kg carcass weight, depending on production practices and system boundaries [128,129].

Emerging technologies are unlocking new possibilities for optimised mushroom production and biofortification. AI-driven monitoring systems can adjust micronutrient dosing in real-time to maximise uptake. Such innovations demonstrate how mushroom cultivation can serve as a platform for food system transformation, supporting SDG 17 (Partnerships for the Goals) through interdisciplinary collaboration. To fully realise this potential, coordinated policy action is needed. Governments should incentivise urban vertical farming initiatives through targeted subsidies and tax incentives, while regulatory bodies must streamline approval processes for novel food products and ingredients derived from biofortified mushrooms. Research institutions and private sector partners should collaborate to scale these technologies, particularly in food-insecure regions.

When implemented within controlled environment systems, biofortified *Pleurotus* production represents a triple-win solution: delivering nutritionally dense food with minimal environmental impact while creating economic opportunities in both developed and developing contexts. As we approach the 2030 deadline for the SDGs, such innovative approaches to sustainable food production will be crucial for building resilient food systems that can nourish a growing global population within planetary boundaries.

## 8. Conclusions and Future Perspectives

This review highlights the potential of *Pleurotus* spp. as sustainable protein sources to address growing global challenges related to food security, environmental degradation, and nutrient deficiencies. Their ability to grow on a wide range of agro-industrial bioresidues contributes to waste valorisation and supports circular bioeconomy strategies. Notably, *P. ostreatus* has demonstrated high biological efficiency under different substrate conditions, while *Pleurotus* species overall exhibit relevant protein contents, ranging from 12.3% to 34.7% DW depending on species and substrate composition, with *P. citrinopileatus* showing the highest reported values.

Biofortification studies have demonstrated that *Pleurotus* spp. are capable of accumulating significant concentrations of Se and Fe under controlled cultivation conditions using defined mineral supplementation strategies. For example, Se accumulation reached up to 858 µg/g in *P. ostreatus* supplemented with 102 mg/kg Se(IV), while Fe concentrations peaked at 4176 µg/g in *P. pulmonarius* using FeSO_4_. However, the nutritional relevance of these enrichments depends not only on total accumulation but also on mineral speciation, bioaccessibility, and safe intake levels, which remain insufficiently investigated.

Despite the promising results reported across studies, substantial methodological heterogeneity remains a major limitation, particularly regarding substrate composition, mineral sources, supplementation levels, and cultivation conditions. Future research should therefore prioritise the standardisation of cultivation and biofortification protocols, as well as the evaluation of mineral speciation, bioaccessibility, bioavailability, and long-term safety through in vitro and in vivo studies. Additional efforts are also needed to better understand consumer acceptance, technological applicability, and scalability under commercial production systems. In summary, *Pleurotus* mushrooms function as a viable protein alternative and potential carriers of essential minerals when subjected to controlled biofortification strategies, representing a promising area for research and technological development with potential relevance for future nutritional and public health strategies.

## Figures and Tables

**Table 2:** Cultivation parameters, biological efficiency, and protein content of *Pleurotus* spp. under different substrates.

*Pleurotus* Species	Substrate	Incubation		Fruiting			Biological Efficiency (%)	Protein Content (% DW ^1^/FW ^2^)	Reference
		Temperature	Humidity	Temperature	Humidity	Light Intensity			
*P. eryngii*	Korshinsk peashrub, sugarcane bagasse, and sawdust	25 ± 1 °C	62%	16 ± 2 °C	80–90%	1500–2000 lux	59 ± 4–71 ± 5	15.11 ± 0.01–23.72 ± 0.02/-	[75,76]
*P. eryngii*	Sawdust and rice straw	25 ± 2 °C	65%	13–22 °C	70–85%	180–250 lux	46.8–73.5	-	[71]
*P. eryngii*	*G. biloba* leaf, sawdust, sugarcane bagasse, and a mix of bioresidues	24 ± 1 °C	60%	10–13 °C	90%	1500–2000 lux	74 ± 11–87 ± 10	17.60 ± 0.00–22.00 ± 0.06/-	[74]
*P. eryngii*	Spent substrates and waste products of mushroom cultivation	26 ± 1 °C	80%	16 ± 1 °C	95%	700 lux	53 ± 14–121 ± 7	-	[57]
*P. djamor*	Sawdust and rice bran	25 ± 2 °C	60%	25 ± 2 °C	95–99%	50–100 lux	92 ± 9	26.0 ± 0.2/-	[77]
*P. djamor*	Bagasse of the *A. salmiana* shoots and wheat grains	24–26 °C	70–80%	18–20 °C	80–90%	-	58 ± 9–71 ± 10	15.20 ± 0–26.3 ± 0.7/-	[79,80]
*P. djamor*	Agave bagasse and barley straw	25 ± 5 °C	50–70%	-	70%	Dark	37 ± 1–57 ± 1	-/6.5 ± 0.1–2.7 ± 0.4	[78]
*P. citrinopileatus*	Spent mushroom substrate, hydroponic roots of leafy vegetables, and wheat straw	24 ± 1 °C	75–80%	17 ± 1 °C	85%	-	39 ± 3–74 ± 5	24 ± 1–34.7 ± 0.8/-	[81]

^1^ DW: dry weight; ^2^ FW: fresh weight.

## Data Availability

No new data were created or analysed in this study. Data sharing is not applicable to this article.
